# Cytokine Toxicity and Bacterial Dysbiosis in Chemotherapy- and/or Radiotherapy-Induced Oral Mucositis: Pathophysiological Mechanisms and Therapeutic Interventions

**DOI:** 10.3390/life16040644

**Published:** 2026-04-11

**Authors:** Pouria Abdolmohammadi, Maral Aali, Christian Lehmann

**Affiliations:** 1 Department of Microbiology and Immunology, Dalhousie University, Halifax, NS B2H 0A3, Canada; pouria.abdolmohammadi@dal.ca; 2 Division of Otolaryngology—Head and Neck Surgery, Department of Surgery, Dalhousie University, Halifax, NS B3H 1Y9, Canada; m.aali@dal.ca; 3 Department of Anesthesia, Pain Management and Perioperative Medicine, Dalhousie University, Halifax, NS B2H 0A3, Canada

**Keywords:** head and neck cancer, oral mucositis, chemotherapy, radiotherapy, cytokine, cytokine toxicity, anti-inflammatory, microbiome, bacterial dysbiosis, oral microbiota manipulation

## Abstract

Chemotherapy- and/or radiotherapy-induced oral mucositis (CRIOM) is a common complication in patients with head and neck cancer, driven largely by excessive proinflammatory cytokine signalling and treatment-associated bacterial dysbiosis. This narrative review synthesizes current mechanistic evidence and summarizes emerging therapeutic strategies targeting these pathways. Research indicates that elevated levels of IL-1β, IL-6, TNF, iNOS, and nitric oxide amplify tissue injury and ulceration, while disruption of oral and gut microbial communities, characterized by loss of beneficial commensals and enrichment of pathogenic taxa, further exacerbates mucosal inflammation. Anti-inflammatory agents, including pentoxifylline, atorvastatin, trans-caryophyllene, azilsartan, recombinant human IL-11, and low-level laser therapy have been shown in preclinical models to reduce cytokine levels and promote mucosal healing. Similarly, microbiome-targeted approaches, such as oral microbiota transplantation and multi-strain probiotic formulations, have demonstrated potential in restoring microbial balance and attenuating CRIOM severity, with current evidence including both preclinical and clinical studies. Overall, current findings highlight cytokine toxicity and dysbiosis as synergistic drivers of CRIOM and support anti-inflammatory and microbiome-modulating strategies as promising adjunctive approaches; however, further well-designed clinical studies are required to validate their efficacy and guide clinical translation.

## 1. Introduction

**Figure 1:**
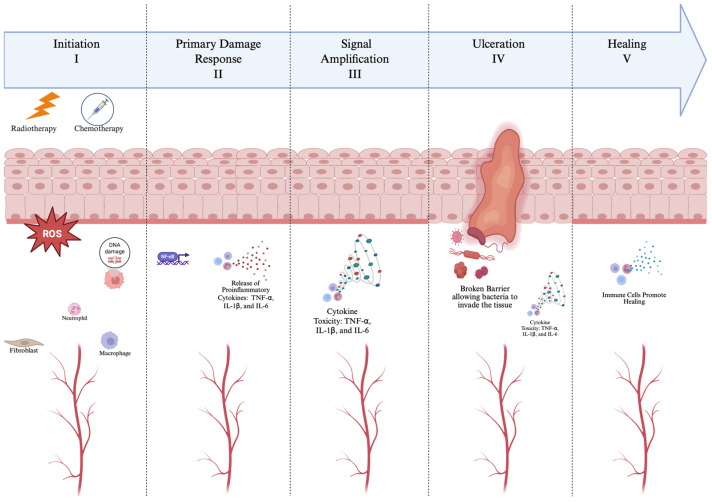
Conceptual representation of the five-phase biological progression of chemotherapy- and/or radiotherapy-induced oral mucositis. In Phase I (Initiation), radiotherapy and chemotherapy generate reactive oxygen species (ROS), causing DNA damage and activating innate immune cells. Phase II (Primary Damage Response) involves NF-κB activation and the release of proinflammatory cytokines such as TNF, IL-1β, and IL-6. In Phase III (Signal Amplification), cytokine toxicity intensifies inflammation and tissue injury through positive feedback mechanisms. Phase IV (Ulceration) is characterized by epithelial breakdown and bacterial invasion, further exacerbating mucosal damage. Finally, in Phase V (Healing), anti-inflammatory cytokines and growth factors promote epithelial regeneration and mucosal restoration. Created in BioRender. Zhou, J. (2026) https://BioRender.com/ltvdniq (accessed on 2 November 2025).

Head and neck cancer remains a significant global health burden, ranking as the seventh most common cancer worldwide [1]. According to GLOBOCAN 2022, approximately 940,000 new cases and 480,000 deaths were reported globally, accounting for 4.7% of all cancer cases and 4.9% of cancer-related mortality [1]. The incidence and mortality of head and neck cancer show substantial geographic and socioeconomic disparities, with higher burdens observed in South Central Asia and Eastern Europe. Notably, men exhibit a 2–4-fold higher incidence and mortality compared to women, and the disease predominantly affects older populations, with peak incidence observed after the age of 50. Despite some declines in incidence and mortality rates in certain regions, the global burden of head and neck cancer is projected to increase substantially, potentially reaching over 1.5 million new cases and more than 800,000 deaths annually by 2050 if current trends persist. The majority of head and neck cancers are squamous cell carcinomas, originating from the epithelial lining of the oral cavity, pharynx, and larynx [2]. Oral radiotherapy, often combined with chemotherapy, is one of the most prevalent and effective strategies used for the treatment of head and neck cancer [3]. However, this treatment is harmful to the normal tissue of the oral cavity and upper gastrointestinal tract, frequently resulting in oral mucositis: a common side effect and potentially severe complication [3]. As shown in Figure 1, the pathophysiology of mucositis has been described as a five-phase process [4]. Initially, radiation triggers the production of reactive oxygen species (ROS) in phase 1, leading to the recruitment of innate immunity and the release of proinflammatory cytokines in phase 2. Subsequently, a signal amplification loop occurs in phase 3, leading to further tissue injury, erythema, inflammation, and apoptosis. Phase 4 is characterized by mucosal ulceration and bacterial colonization, often resulting in xerostomia. Finally, phase 5 involves epithelialization and healing.

Oral mucositis clinically manifests as extremely painful lesions in the oral mucosa that are refractory to pain management, due to its multifactorial etiology of both nociceptive and neuropathic mechanisms of injury [5]. These lesions often develop within the first few weeks of radiotherapy or shortly after initiation of cytotoxic chemotherapy and frequently worsen in severity with increasing cumulative treatment dose. In severe cases, patients may develop oral ulcers that cause intractable pain, impede oral intake, delay oncologic therapy, and increase the risk of both local and systemic infections. As oral mucositis progresses, patients experience dysphagia and odynophagia (difficulty and pain with swallowing), dysarthria (slurred speech), and the inability to receive adequate oral nutrition. These symptoms are often exacerbated in head and neck cancer patients by concurrent xerostomia, dysgeusia (taste disturbances), and baseline or treatment-related dysphagia resulting from tumor size and anatomic location. When severe, mucositis can become dose-limiting, leading to treatment interruptions or dose reductions and, in some cases, early cessation of oncologic therapy, which may compromise locoregional control and survival. Thus, oral mucositis contributes significantly to healthcare resource utilization and costs, as it is linked to higher rates of hospital admissions, prolonged inpatient stays, more frequent emergency and outpatient visits, increased nutritional support requirements, and greater reliance on opioid analgesics and antibiotic therapy [6]. Numerous studies have identified cytokine toxicity and bacterial dysbiosis as pivotal factors in the development of chemotherapy- and/or radiotherapy-induced oral mucositis (CRIOM) in head and neck cancer patients, suggesting that therapeutic modulation of these two factors may ameliorate severe oral mucositis [7,8]. Notably, increasing evidence suggests a bidirectional relationship between cytokine-mediated inflammation and microbiome dysbiosis, whereby inflammatory signaling can alter microbial composition, while microbial imbalances can further amplify immune responses and tissue injury. This interplay provides a strong rationale for examining these processes together in the context of CRIOM.

Despite growing evidence on the roles of cytokine-mediated inflammation and microbiome dysbiosis in CRIOM, existing studies have largely examined these mechanisms in isolation, and a comprehensive synthesis integrating these interconnected mechanisms and their therapeutic implications remains limited. This review aims to address this gap by providing an integrated overview of these pathways, elucidating the potential synergistic roles of cytokine toxicity and bacterial dysbiosis, and critically evaluating emerging therapeutic strategies targeting them.

## 2. Materials and Methods

A literature search was conducted to address cytokine toxicity and bacterial dysbiosis as key contributors to the pathogenesis of oral mucositis and to identify studies investigating the anti-inflammatory and microbiome-modulating mechanisms involved in CRIOM, aimed at alleviating the condition. The search was performed using PubMed, employing combinations of keywords and Medical Subject Headings (MeSH), including: “head and neck cancer”, “oral mucositis”, “chemotherapy”, “radiotherapy”, “cytokines”, “cytokine toxicity”, “microbiome”, “bacterial dysbiosis”, “anti-inflammatory”, and “oral microbiota manipulation”. The search included studies published from 1 January 1995 to 23 March 2026, with the final search conducted on 23 March 2026. Studies were included if they were original research articles published in English, investigated CRIOM, and evaluated anti-inflammatory agents or microbiome-targeted interventions. Both preclinical studies (in vivo animal models and in vitro cellular experiments) and clinical studies involving patients were included. Exclusion criteria comprised studies unrelated to oral mucositis, those not addressing inflammatory or microbiome-related mechanisms, and articles without full-text access. The initial search yielded approximately 139 records. After removal of duplicates and screening of titles and abstracts, 89 articles were assessed for eligibility through full-text review. A total of 63 studies were included in the final analysis. Study selection and screening were performed by a single reviewer; therefore, no inter-reviewer disagreement resolution was required. Given the narrative design of this review, a formal duplicate screening process was not applied. In addition, a formal quality assessment or risk of bias evaluation was not conducted, which represents a limitation of this review.

## 3. Cytokine Toxicity: The Overproduction of Proinflammatory Cytokines During CRIOM

The increase in the production of proinflammatory cytokines during chemotherapy and/or radiotherapy in head and neck cancer patients has been known as a central factor for oral mucositis development [9]. While chemotherapy and radiotherapy remain among the most effective therapeutic strategies for eradicating malignant cells and improving patient survival, their cytotoxic mechanisms also affect normal rapidly dividing tissues, such as the oral epithelium [10]. Upon chemotherapy and/or radiotherapy, a massive generation of reactive oxygen species (ROS) occurs, causing the damage of the genetic contents of oral epithelial cells, their death by apoptosis and upregulation of several transcriptional factors such as nuclear factor-kB (NF-kB), contributing to the production of a variety of proinflammatory cytokines [11,12]. This upregulation of inflammatory cytokines during the second phase of chemotherapy or radiotherapy is a key player in causing oral tissue damage seen in phases 3 and 4 [4]. While multiple oral cell types, including epithelial, endothelial, and connective tissue cells, are capable of producing proinflammatory cytokines, mucositis pathobiology involves a coordinated response across these tissues. Evidence suggests that epithelial and submucosal compartments contribute to the initial cytokine release following treatment-induced injury, which is subsequently amplified by infiltrating immune cells, including macrophages and neutrophils, thereby leading to sustained tissue damage and cytokine signaling [9,11]. However, based on the currently available evidence, it is not possible to determine which oral cell population is principally responsible for the observed cytokine elevations, as mucositis pathobiology involves a complex and coordinated interaction across multiple cell types and tissue compartments.

### 3.1. The Major Proinflammatory Cytokines in CRIOM

Saliva is a clinically meaningful fluid that reflects the local mucosal environment, allowing non-invasive monitoring of cytokine modulation during treatment [13]. In a study involving 55 patients with advanced head and neck cancer undergoing chemoradiation therapy, there was a significant rise in proinflammatory cytokines, particularly IL-1β, IL-6, and TNF during chemoradiation therapy with higher levels correlating with more severe mucositis, as compared to the control group. Interestingly, early increase in IL-1β and IL-6 by week 3 was an even stronger predictor of high-grade mucositis, indicating that salivary cytokine profiles can reflect and even anticipate local tissue inflammation and damage. Therefore, the authors confirmed the role of proinflammatory cytokines in the CRIOM severity, in particular that IL-1β and IL-6 exert a stronger devastating effect.

### 3.2. The Upregulation of Proinflammatory Cytokines Triggers the Production of Other Proinflammatory Elements, Deteriorating Oral Mucositis

In another study, the authors evaluated the role of proinflammatory cytokines and other mediators, including inducible nitric oxide synthase (iNOS) and nitric oxide (NO) in CRIOM [14]. The enzyme iNOS plays an essential role in the macrophage inflammatory response by producing NO, which is strongly induced by proinflammatory stimuli [15]. NO functions as a signalling molecule central to inflammation: exerting anti-inflammatory effects under normal conditions but acting as a pro-inflammatory mediator when overproduced [16]. In this study, Cobalt-60 (Co-60) radiation was applied to the hamster cheek pouches to induce oral mucositis [14]. On day 13 of treatment, when myeloperoxidase, an inflammatory marker, peaked, there was also a marked increase in IL-1β, TNF, NO and iNOS, along with a reduction in IL-10 levels, all contributing to mucositis development. On the other hand, treatment with pentoxiphylline (PTX), a methylxanthine derivative known to suppress proinflammatory cytokines and inhibit TNF gene transcription, led to a significant decrease in TNF, IL-1β and iNOS expression, thus preventing Co-60-induced oral mucositis as reflected by lower myeloperoxidase levels. Since PTX partially reduced iNOS, the authors hypothesized that direct iNOS inhibition might further prevent mucositis. Treatment with aminoguanidine (AMG), an iNOS inhibitor, reduced myeloperoxidase expression and lowered TNF and IL-1β levels, alongside decreases in citrulline and nitrite concentrations, though iNOS expression was unchanged. These findings suggest that TNF and IL-1β play a key role in CRIOM pathogenesis, possibly by inducing iNOS and nitric oxide production as a terminal mediator.

### 3.3. The Upregulation of Proinflammatory Cytokines Leads to Dysphagia, Necessitating Installation of a PEG

Oral mucositis can substantially impair patients’ ability to maintain adequate oral intake, and in more severe cases, nutrition must be supported through enteral methods such as nasogastric (NG) or orogastric (OG) tubes, or through percutaneous endoscopic gastrostomy (PEG) placement when feeding difficulties and dysphagia persist [17,18]. Meirovitz et al. (2010) investigated the relationship between serum cytokine levels and severity of mucositis necessitating installation of a PEG tube in head and neck cancer patients undergoing combined chemo-radiation therapy [17]. IL-6 level rose significantly after the second week of the therapy and correlated with PEG tube need: patients requiring PEG tube had a median IL-6 of 5.6 pg/mL, versus 3.2 pg/mL in others. IL-1, TNF and IL-10 did not show any association. Interestingly, IL-8 levels decreased during treatment: a paradox given its proinflammatory role, echoing previous reports [17,19,20]. However, as these studies focused on healthy tissue responses to irradiation, Meirovitz et al. (2010) [17] suggested that IL-8 may help protect against irradiation damage. Consistent with this, another study in patients receiving radiotherapy reported that lower IL-8 levels were associated with an increased risk of radiation-induced tissue injury, supporting a potential context-dependent and protective role of IL-8 in response to irradiation [21]. Overall, elevated IL-6 levels were positively correlated with mucositis severity and dysphagia, leading to the installation of a PEG.

### 3.4. The Strengths, Gaps in the Knowledge and Suggested Improvements

Although the current studies highlight the key role of proinflammatory cytokine overproduction in oral mucositis, important knowledge gaps persist regarding its pathogenesis in head and neck cancer patients receiving chemo- or radiotherapy. Two important factors missing among these articles are the effects of sex and age on proinflammatory cytokine production contributing to oral mucositis. The effects of sex and age have been confirmed for other oral inflammatory diseases, such as periodontal disease [22]. Similarly, sex appears to influence susceptibility to oral mucosal injury, with evidence showing that women have a higher likelihood of developing severe oral mucositis during intensive treatments such as autologous stem cell transplantation. This difference has been linked to sex-related variations in immune responses, hormonal influences on mucosal integrity and inflammatory regulation, and differences in epithelial turnover, all of which may increase vulnerability to mucosal damage [23]. In addition, variability across studies, including heterogeneity in treatment regimens (e.g., differences in chemotherapy agents, radiation doses, and combined treatment protocols) and cytokine measurement methodologies (e.g., sample source, assay type, and timing of collection), may contribute to inconsistencies in reported findings and should be considered when interpreting the available evidence. Another limitation of the included studies is that they do not identify which oral cell populations are responsible for the cytokine elevations they report. In reality, multiple cell types, including oral epithelial cells, resident immune cells such as macrophages and dendritic cells, and stromal cells such as fibroblasts, can all produce pro-inflammatory cytokines during mucosal injury [24]. The other considerable issue by the authors is not investigating the mechanisms of action involved in the upregulation of proinflammatory cytokines. Knowing the mechanisms of action is also important for prevention and treatment purposes. The proteins involved in the pathways of proinflammatory cytokine production can be feasible targets for the inhibition of proinflammatory cytokines.

## 4. The Alleviation of Oral Mucositis Using Anti-Inflammatory Approaches

**Figure 2:**
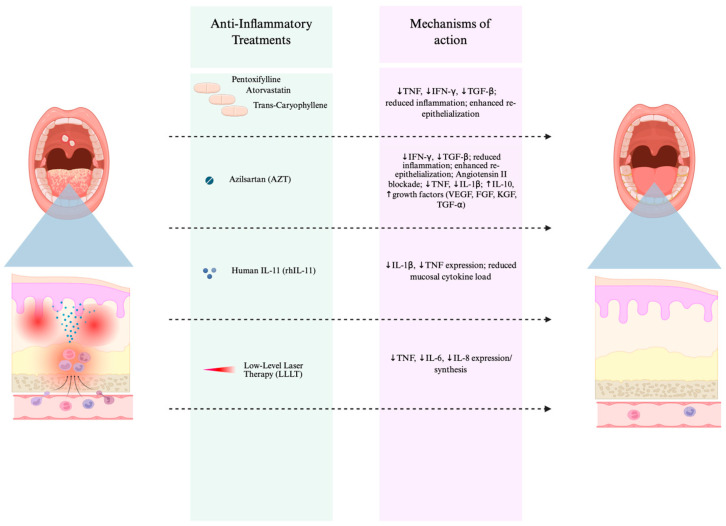
Conceptual overview of anti-inflammatory therapeutic strategies and their mechanisms of action in alleviating chemotherapy- and/or radiotherapy-induced oral mucositis. Pentoxifylline, atorvastatin, and trans-caryophyllene suppress TNF, IFN-γ, and TGF-β to reduce inflammation and enhance re-epithelialization. Azilsartan (AZT) blocks angiotensin II signalling, decreases TNF and IL-1β, increases IL-10, and upregulates growth factors such as VEGF, FGF, KGF, and TGF-α to accelerate tissue repair. Recombinant human IL-11 (rhIL-11) downregulates IL-1β and TNF to lower mucosal cytokine load and preserve epithelial integrity. Low-level laser therapy (LLLT) further decreases TNF, IL-6, and IL-8 expression, exerting biomodulatory effects that reduce inflammation and promote mucosal healing. Created in BioRender. Zhou, J. (2026) https://BioRender.com/suvb78i (accessed on 2 November 2025).

The study of inflammatory processes in CRIOM enables the development of targeted, biology-based strategies for the modulation of oral mucositis [25]. Anti-inflammatory interventions remain a promising strategy for their prevention and treatment [26], as inhibiting proinflammatory cytokines such as TNF and IL-1β has been shown to alleviate CRIOM [27,28,29]. However, since inflammatory signalling also contributes to the therapeutic effects of chemo- and radio-therapy, excessive suppression can impair cancer cell killing. Recent studies suggest that mechanism-based therapies, illustrated in Figure 2, can alleviate mucositis without compromising anticancer efficacy, as some anti-inflammatory or antioxidant agents selectively protect normal mucosa while preserving tumor sensitivity to treatment-induced apoptosis. This selectivity supports the development of safe adjunctive therapies for mucositis management that do not interfere with chemotherapy or radiation outcomes [30]. It is important to consider the potential oncologic implications of cytokine-targeted anti-inflammatory strategies. Tumor necrosis factor (TNF) plays a context-dependent role in cancer biology, contributing to both tumor-promoting inflammation and immune-mediated tumor surveillance. Notably, clinical data from patients receiving TNF inhibitors have not demonstrated an increased overall risk of malignancy or solid tumors, although long-term data remain limited and certain cancer subtypes require further investigation [31]. Similarly, emerging evidence suggests that targeted cytokine modulation, such as interleukin-6 (IL-6) blockade, may reduce inflammatory toxicity without impairing—and potentially even enhancing—anti-tumor immune responses. Preclinical and clinical observations indicate that IL-6 inhibition can preserve or augment T cell–mediated tumor control while mitigating inflammatory complications. Nevertheless, given the complex and context-dependent roles of inflammatory cytokines in tumor biology, further clinical studies are required to fully establish the long-term oncologic safety and immunological consequences of cytokine-suppressive therapies in this setting [32].

### 4.1. The Anti-Inflammatory Effects of the Drugs Pentoxifylline, Atorvastatin, and Trans-Caryophyllene Contribute to the Alleviation of Oral Mucositis

Oral mucositis, characterized by epithelial breakdown, ulceration, and inflammatory cell infiltration, is driven by cytokines such as TNF, IFN-γ, and TGF-β [33]. Pentoxifylline, atorvastatin, and trans-caryophyllene have been investigated for their preventive potential by suppressing TNF and IFN-γ expression, regulating nitric oxide production, and promoting epithelial recovery [33]. In a 5-fluorouracil-induced oral mucositis model in male Wistar rats, these agents reduced cytokine (TNF, IFN-γ, and TGF-β) expression, decreased ulceration, and enhanced re-epithelialization [33]. Preventative atorvastatin therapy significantly lowered TNF serum levels as compared to control and other experimental groups, while trans-caryophyllene treatment reduced in situ TNF expression and inflammation, supporting healing. Systemically, trans-caryophyllene modulated IFN-γ levels during the onset and healing stages of oral mucositis (Days 8 and 15 of the experiment, respectively), indicating that this cytokine does not directly influence the worsening (Day 11) of the ulcers. Pentoxifylline also showed significant effects during ulcer remission (Day 15). Atorvastatin reduced systemic levels of TNF and INF-γ across all stages. Histologically, trans-caryophyllene prevented oral wounds and atorvastatin improved surface healing, suggesting that both agents mitigate oral mucositis by lowering proinflammatory mediators.

### 4.2. The Drug Azilsartan (AZT) Helps Alleviate Oral Mucositis Through Its Anti-Inflammatory Effects

Azilsartan (AZT), an angiotensin II receptor blocker with anti-inflammatory and tissue-protective properties, was investigated for its potential to mitigate CRIOM [34]. The drug modulates cytokines by reducing TNF-α and IL-1β, increasing IL-10, and enhancing growth factors such as VEGF and FGF, thereby promoting epithelial repair and limiting mucosal inflammation. Using a 5-fluorouracil-induced oral mucositis model in Syrian hamsters, the study evaluated the effects of AZT on mucosal injury and healing [34]. They used macroscopic analysis, and cheek pouch samples were removed for histopathologic analysis. In this study, TNF, IL-1β, IL-10, VEGF, FGF, KGF and TGF-α levels were measured using ELISA and immunohistochemical analysis. The results revealed a significant decrease in TNF and IL-1β upon oral treatment with 1 mg/kg AZT. Furthermore, the AZT1/5-FU group showed dramatically increased levels of IL-10 and TGF-α compared to the 5-FU/saline group. The most effective dose for the AZT efficacy was 1 mg/kg, in which AZT generally demonstrated effectiveness regardless of dose, with a lower dose being preferred for achieving the optimal clinical outcomes by speeding up the healing process. In addition, the findings illustrated that AZT sped up the healing process in an experimental model of 5-FU-induced oral mucositis by promoting granulation tissue formation, the migration of fibroblasts and keratinocytes, and collagen deposition. These results were supported by the elevated levels of TGF-α, FGF, and KGF, along with the upregulation of VEGF, which has been linked to angiogenesis in scar tissue. Together, administration of AZT at a dose of 1 mg/kg sped up the healing process by stimulating growth factors that are critical for angiogenesis and re-epithelialization, along with increasing IL-10 levels.

### 4.3. The Mechanisms by Which Recombinant Human Interleukin-11 Modulates the Progression of Radiation-Induced Oral Mucositis

Interleukin-11 (IL-11), a cytokine with cytoprotective and anti-inflammatory properties, was investigated for its ability to mitigate radiation-induced oral mucositis [35]. IL-11 downregulates pro-inflammatory cytokines such as IL-1β and TNF-α, protects connective tissue, and promotes epithelial proliferation and differentiation, thereby reducing inflammation and preserving mucosal integrity. Using a model of acute radiation injury in male golden Syrian hamsters, recombinant human IL-11 (rhIL-11) was evaluated for its effects on mucosal cytokine expression, apoptosis, and histological alterations [35]. According to the mucositis scores, there was an increased mucositis severity at day 15; however, animals treated with rhIL-11 showed less mucositis compared to placebo controls, suggesting the first evidence that rhIL-11 can modulate radiation-induced mucositis. This study observed that local tissue levels of IL-1β and TNF significantly increased after radiation, and this rise in both cytokines paralleled the progression of mucositis. However, a significant decrease in IL-1β levels, as detected by immunohistochemical and PCR techniques, was observed in rhIL-11-treated animals. This reduction was associated with a lower severity of mucositis. Immunohistochemical analysis indicated that the decrease in IL-1β levels was not caused by a reduction in the intensity of the inflammatory infiltrate, but rather due to lower expression of the cytokine by submucosal inflammatory cells. Moreover, RNA levels of TNF and IL-1β were lower in rhIL-11-treated hamsters. Unlike IL-1β and TNF, there were no differences in IL-2 or TGF-β levels between the control and rhIL-11-treated hamsters. This observation implies a significant connection between elevated IL-1β and TNF levels and the development of mucositis, supporting the concept that rhIL-11 activity might partially stem from its ability to suppress the expression of these proinflammatory cytokines.

### 4.4. Biomodulation of Inflammatory Cytokines Associated with Oral Mucositis by Low-Level Laser Therapy

Low-level laser therapy (LLLT) was investigated for its potential to modulate inflammatory cytokine expression associated with oral mucositis [36]. LLLT has been shown to promote wound healing by enhancing cell proliferation, migration, and growth factor expression, while also reducing inflammatory mediator release [36]. Because inflammatory cytokines such as TNF, IL-6, and IL-8 play a central role in the onset and severity of mucositis, this study evaluated the biomodulatory effects of LLLT on their gene and protein expression in lipopolysaccharide-stimulated human gingival fibroblasts [36]. In this study, primary gingival fibroblasts were treated with LPS and exposed to LLLT irradiation at 0, 0.5, 1.5 or 3 J cm^−2^. The gene expression levels of TNF, IL-1β, IL-6, and IL-8 were assessed using Real-Time PCR, and the protein synthesis of these cytokines was measured using ELISA. According to the results, non-irradiated gingival fibroblasts exposed to LPS exhibited an increased expression of all cytokines studied, except for IL-1β, compared to cells not exposed to LPS. Irradiation following LPS treatment at energy densities of 1.5 J/cm^2^ and 3 J/cm^2^ reduced the expression of TNF, IL-6, and IL-8. Although 0.5 J/cm^2^ was not effective in modulating the gene expression of these inflammatory cytokines. Regarding the results obtained by ELISA, low-level laser therapy reduced TNF synthesis at all energy densities, while IL-6 synthesis was decreased at 1.5 J/cm^2^ and 3 J/cm^2^. However, the therapy did not affect the synthesis of IL-1β and IL-8. These results showed that LLLT had a beneficial biomodulatory impact on the expression of inflammatory cytokines related to oral mucositis in human gingival fibroblasts.

Low-level laser therapy (LLLT) has been incorporated into current supportive care strategies for oral mucositis, with clinical guidelines (e.g., MASCC/ISOO) recommending its use in specific settings, particularly in patients undergoing high-dose chemotherapy and hematopoietic stem cell transplantation, where evidence is strongest [37]. While randomized clinical trials and translational studies have demonstrated its efficacy in reducing the incidence, severity, and duration of oral mucositis, particularly in head and neck cancer patients, variability in treatment protocols and limited standardization across studies continue to limit its broader clinical adoption [38]. Accordingly, LLLT is currently considered a promising adjunctive modality within established oral mucositis management strategies rather than a universally standardized treatment.

### 4.5. The Strengths, Gaps in the Knowledge, and Suggested Improvements

The primary strength of the presented articles is the strong data showing that modulating the production of proinflammatory cytokines can attenuate CRIOM, as summarized in Table 1. Therefore, their findings may be promising adjunctive treatments for oral mucositis. However, some weaknesses are evident in the articles, particularly their lack of consideration for the side effects of the drugs used, which is essential for determining whether these agents can be safely applied for anti-inflammatory management of oral mucositis. They could have compared cytokine levels after anti-inflammatory treatment with a normal range that an individual’s body needs to function properly. They could also monitor for the occurrence of immunosuppressive diseases upon drug treatment. The other considerable issue is that the authors did not consider the effects of sex and age in their results. Finally, the articles presented in Section 3.1, Section 3.2 and Section 3.3 were animal in vivo, and the article presented in Section 3.4 was an in vitro human model, so there is a need to investigate how the treatment would work for an in vivo human model.

## 5. The Role of Oral Microbiota in CRIOM

The commensal microbiota can enormously impact the colonization and resistance of pathogenic microorganisms and stimulate primary immunity [39]. Upon oral microbiota dysbiosis, the oral mucosal epithelial defense can be compromised, thus accelerating the pathological processes [39]. Exposure to cytotoxic cancer treatments such as chemotherapy and/or radiotherapy is linked to significant alterations in the oral microbiome. However, distinguishing between cause and effect has been extremely challenging [40]. Several studies have documented that the alterations in the oral microbiome after exposure to chemotherapy- and/or radiotherapy contribute to the development of oral mucositis [40]. Recent research indicates that improved outcomes for oral mucositis are linked to a more resilient oral microbiome that remains stable upon chemotherapy- and/or radiotherapy treatments [41,42]. Furthermore, during the fourth stage of the mucositis, the ulceration phase, the breaks in the submucosa permit some microorganisms, typically symbiotic inhabitants of healthy mucosa, to invade this tissue, leading to an inflammation response mediated by mononuclear infiltrating cells, hence promoting new pro-inflammatory cytokines release that can amplify expression of pro-apoptotic mediators and increase tissue damage [43]. It is important to note that much of the current evidence linking microbiome alterations to CRIOM severity is based on associative findings rather than direct causative relationships. While dysbiosis has been correlated with disease progression and severity, a causal role has not been definitively established. In addition, several confounding factors, including antibiotic use, proton pump inhibitors, dietary habits, and oral hygiene, may significantly influence microbiome composition and should be carefully considered when interpreting these findings. Therefore, further well-controlled studies are required to clarify the causal relationship between microbiome dysbiosis and CRIOM.

### 5.1. Oral Dysbiosis Has a Significant Detrimental Impact on the Severity of Oral Mucositis

In a study, the authors determined whether the commensal microbiota can influence the severity of CRIOM [44]. In this study, specific-pathogen-free (SPF) and germ-free Swiss Webster mice in the experimental groups were administered 5-Fluorouracil (5-FU) to induce oral mucositis. Then, differences in epithelial thickness, cell proliferation/turnover rates, and expression levels of metalloproteinases and pro-inflammatory mediators were analyzed using histopathological and immunohistochemical tests. The oral cavities of germ-free mice are devoid of any microorganisms. In contrast, SPF mice harbour bacteria from the genera *Streptococcus*, *Lactobacillus*, *Staphylococcus*, *Enterococcus* and *Propionibacterium*. The results revealed that 5-FU-treated SPF mice exhibited characteristic histopathological features of oral mucositis, with a significant reduction in the proliferation of oral mucosal cells compared to the other groups. The qualitative analysis revealed that the oral epithelium was dramatically thinner in 5-FU-treated SPF mice compared to 5-FU-treated germ-free mice. Histological grading showed that the severity of oral mucositis was grade 3 in SPF mice and grade 1 in germ-free mice. The levels of proinflammatory cytokines, such as IL-1β, TNF, and MPO, were significantly higher in the oral mucosa of SPF mice compared to germ-free mice. They also discovered that the proinflammatory cytokines IL-1β, TNF and MPO are expressed by cells in the oral epithelium, endothelium, and mucosal connective tissues in 5-FU-treated SPF mice. They detected increased levels of MPO in the connective tissues of the oral mucosa in 5-FU-treated SPF mice. MPO, released from neutrophil’s azurophilic granules, facilitates the production of hypochlorous acid, leading to oxidative damage to host tissue. In addition, they observed elevated levels of expression of MMP-3 and -9 in the basal epithelium, lamina propria and submucosa of 5-FU-treated SPF mice. MMP-3 may contribute to mucosal damage by disrupting cell-to-cell and cell-to-extracellular matrix (ECM) connections through the degradation of collagens, fibronectin, laminin, aggrecan, insulin-like growth factor binding protein-3, and serpins [44]. As tissue injury advances during the development of oral mucositis, the tight junction proteins such as E-cadherin, occludin and claudin-1 are downregulated, which can further weaken the barrier function of oral mucosa [44]. This increased permeability allows bacterial products to pass through, resulting in further tissue damage [44]. Hence, upon tissue damage, both pathogen-associated molecular patterns (PAMPs) generated by bacterial products and damage-associated molecular patterns (DAMPs) resulting from cellular stress and tissue injury are recognized by the oral epithelium, fibroblasts, and endothelial cells via their pattern recognition receptors (PRRs), including toll-like receptor (TLR)-2, -4, -9, and nucleotide-binding oligomerization domain-containing protein-1 (NOD-1) and -2, resulting in intensified inflammatory responses [44]. Together, comparing the 5-FU-treated SPF and germ-free mice, the germ-free mice showed less severe oral mucositis.

### 5.2. Chemotherapy-Induced Oral Mucositis Is Associated with Bacterial Dysbiosis

In the study performed by Hong et al. (2019) [45], the authors demonstrated that chemotherapy-induced oral mucositis is associated with bacterial dysbiosis and suggested that these dysbiotic changes can worsen chemotherapy-induced epithelial injury. In this study, they used 49 subjects receiving 5-fluorouracil (5-FU) or doxorubicin-based chemotherapy, and 30 non-cancer subjects, as a control for microbiome stability, then evaluated them longitudinally during one cycle [45]. They also conducted in vitro assays to assess the antibacterial potential of 5-FU on oral microorganisms and its interaction with commensal bacteria in oral epithelial tissues. The results revealed that during chemotherapy, the oral bacteriome experienced significant disruptions. They included that while antibiotic and acid inhibitor intake played a role in these changes, disruptions were also linked to antineoplastics and were independently and significantly associated with the severity of oral mucositis. They found that the changes in the bacteria associated with the mucositis involved a reduction in common health-associated commensals from genera like *Streptococcus*, *Actinomyces*, *Gemella*, *Granulicatella*, and *Veillonella*. However, there was an increase in some Gram-negative bacteria, such as *Fusobacterium nucleatum* and *Prevotella oris.* Also, during chemotherapy, transcriptional responses showed an increase in the expression of genes involved in innate immunity and apoptosis. Finally, using a multilayer epithelial construct, they demonstrated that dysbiotic shifts associated with mucositis may worsen mucosal damage. This is because the mucositis-depleted *Streptococcus salivarius* was tolerated as a commensal, whereas the mucositis-enriched *F. nucleatum* exhibited pro-inflammatory and pro-apoptotic properties [45]. All in all, this study illustrated that chemotherapy-induced oral mucositis is linked to bacterial dysbiosis and shows that dysbiotic shifts can potentially worsen epithelial injury caused by antineoplastic treatments.

### 5.3. Bacterial Colonization and Gene Expression Vary During Different Stages of Mucositis

In one study, the authors assessed the bacterial colonization and evaluated the genes’ expression of MCR-1 (mobilized colistin resistance), VIM2 (β-lactam resistance), TET(K) (tetracycline resistance) and blaKPC (carbapenem resistance) at 3 time points (onset, during, and at the end), among 24 oral mucositis patients with oral cancers undergoing radiotherapy and concomitant radiochemotherapy [46]. The results revealed that the facultative anaerobes isolated from saliva included *Staphylococcus aureus* (22%, *n* = 16), *Staphylococcus epidermidis* (29%, *n* = 21), *Pseudomonas aeruginosa* (28%, *n* = 20), *Escherichia coli* (25%, *n* = 18), and *Klebsiella pneumoniae* (26%, *n* = 19). In the study groups, subjects treated with chemoradiation exhibited higher prevalence rates of *S. aureus* (50%, *n* = 6), *P. aeruginosa* (41.7%, *n* = 5), and *S. epidermidis* (33.3%, *n* = 4), whereas *E. coli* (16.7%, *n* = 2) was the least prevalent by the end of the 6th week of therapy. The Mann–Whitney U test indicated no statistically significant difference in the presence of organisms between the study groups at the 1st, 3rd week, and at the end of therapy. The bacterial isolates collected during and at the end of therapy exhibited higher levels of antibiotic-resistance genes (VIM2, MCR-1, TET(K), bla_KPC_) compared to those collected at the start of the therapy. Together, Bacterial colonization and gene expression varied throughout the different stages of oral mucositis.

### 5.4. The Strengths, Gaps in the Knowledge, and Suggested Improvements

The primary merit among the presented articles is identifying some of the bacteria that experience a change in their population due to CRIOM. So, by knowing how a change in the population of bacteria would affect the severity of the oral mucositis, it is possible to manage the severity of the oral mucositis accordingly. However, the articles have several flaws. One is that the authors did not examine whether there is a sex and age difference in the microbiome and microbiota dysbiosis. It could be an important clue whether male and female patients should be treated differently. Also, in the experiment by Subramaniam & Muthukrishnan (2019) [46], the authors should have examined precisely how the development of antibiotic resistance impacts the severity of the CRIOM. They could have compared the severity of the oral mucositis according to each individual’s antibiotic resistance. Therefore, it could aid in identifying a drug targeting antibiotic resistance genes that would provide the most protective results for the amelioration of oral mucositis. Finally, it is important to consider that several confounding variables, including antibiotic use, proton pump inhibitors, dietary factors, and oral hygiene practices, may significantly influence microbiome composition and inflammatory responses, while methodological variability across studies, including differences in sampling, sequencing platforms, and analytical pipelines, may further contribute to heterogeneity in reported findings, thereby limiting the interpretation of the relationship between microbiome alterations and CRIOM.

## 6. The Manipulation of Oral Microbiota for the Management of Oral Mucositis

**Figure 3:**
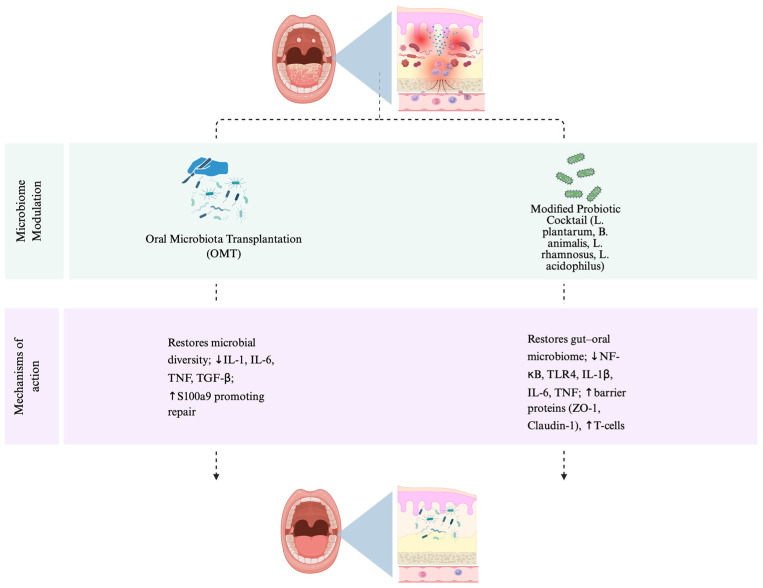
Conceptual overview of microbiome-modulating therapeutic approaches and their mechanisms of action in alleviating chemotherapy- and/or radiotherapy-induced oral mucositis. The figure illustrates two interventions: oral microbiota transplantation (OMT), which restores microbial diversity, decreases IL-1, IL-6, TNF, and TGF-β levels, and upregulates S100a9 expression to promote mucosal repair; and a modified probiotic cocktail containing *Lactobacillus plantarum*, *Bifidobacterium animalis*, *Lactobacillus rhamnosus*, and *Lactobacillus acidophilus*, which restores the gut–oral microbiome balance, suppresses NF-κB, TLR4, IL-1β, IL-6, and TNF expression, enhances epithelial barrier proteins (ZO-1, Claudin-1), and increases T-cell levels. Together, these microbiome-targeted therapies mitigate inflammation, improve epithelial integrity, and promote recovery from oral mucositis. Created in BioRender. Zhou, J. (2026) https://BioRender.com/5oggw05 (accessed on 2 November 2025).

Understanding the role of evolving polymicrobial oral communities in causing or worsening cancer therapy-induced oral mucositis is essential to preventing the severe negative effects associated with CRIOM [47]. On the other hand, the composition of gut microbiota may change during the treatment, which could also influence the severity of the CRIOM in head and neck cancer patients [48]. Proinflammatory gut microbes, whether presented at the baseline or increased after chemotherapy and/or radiotherapy, can worsen inflammatory processes in the oral cavity, leading to severe oral mucositis. This could be due to the changes in the intestinal environment, like inflammation and disruption of the intestinal barrier, or the systemic effects of the gut microbiota on the immune system [49]. Although gut microbes with anti-inflammatory properties can beneficially impact oral mucositis by maintaining intestinal balance and reducing systemic inflammatory signals [49]. Consequently, the modification of gut microbiota has attracted considerable interest among clinicians and scientists as a viable method for alleviating CRIOM in head and neck cancer patients [50,51]. These therapeutic concepts are further summarized in Figure 3, which outlines microbiome-modulating approaches and their mechanisms of action in alleviating chemotherapy- and/or radiotherapy-induced oral mucositis.

### 6.1. Oral Microbiota Transplantation Is a Feasible Approach to Fighting Against Radiotherapy-Induced Oral Mucositis

In a study by Xiao et al. (2021) [52], the authors found that by performing oral microbiota transplantation (OMT), it is possible to fight against head and neck radiotherapy-induced oral mucositis in mice. Fractionated radiation was administered to simulate radiotherapy for nasal, oral and laryngeal cancers (NOALCs) in mouse models [52]. They also recruited 44 nasal, oral and laryngeal cancer patients and compared *Lactobacillaceae* levels during radiotherapy. They analyzed the oral bacterial composition of healthy mouse donors, finding *Streptococcus* and *Rodentibacter* to be the most abundant. They transplanted oral microbiomes from healthy mouse donors to mice that were exposed to local head and neck irradiation. The results revealed that OMT ameliorated irradiation-induced oral mucositis. Hematoxylin and eosin staining showed thinning epithelium and flattened tongue papillae after radiation exposure. However, OMT ameliorated the injuries. The proinflammatory cytokines, IL-1, IL-6, TNF and TGFβ in tongue tissues and plasma could be reduced by OMT. To understand how OMT reduces radiation-induced oral mucositis, they analyzed the oral bacteria in mice after head and neck irradiation, with and without OMT treatment. The results revealed that irradiation did not change the ɑ-diversity of the oral bacteria, but 10 days of OMT increased the ɑ-diversity. Also, OMT reversed the cumulative increase in β-diversity caused by irradiation. Based on 16S rRNA gene sequencing among clinical samples, they observed that radiotherapy to NOALC patients or total head irradiation in mice raised *Lactobacillaceae* levels; however, OMT reversed this change. Suggesting that *Lactobacillaceae* might play a key role in the development of radiation-induced oral mucositis; however, it can be counteracted by OMT. Collectively, their findings indicated that OMT alters the oral bacterial composition to combat radiation-induced dysbiosis and mucositis in the oral cavity. Finally, to further investigate the underlying mechanism by which OMT protects against radiation-induced oral mucositis, they used high-throughput sequencing to assess gene expression in tongue tissues from irradiated mice with and without OMT. The most significant response was from S100a9, which was downregulated after irradiation and upregulated by OMT. Silencing S100a9 negated OMT’s protective effects, worsening symptoms and increasing inflammatory factors, suggesting S100a9’s role in OMT-mediated recovery.

### 6.2. The Use of Modified Probiotic Cocktail Can Significantly Diminish the Severity of Oral Mucositis

In a study, the authors determined the protective effects of probiotic cocktail treatment on chemoradiotherapy (CCRT)-induced oral mucositis in nasopharyngeal cancer (NPC) patients and rat model [53]. The results revealed that providing patients with a probiotic cocktail of *Lactobacillus plantarum*, *Bifidobacterium animalis*, *Lactobacillus rhamnosus*, and *Lactobacillus acidophilus* had a protective effect against chemoradiotherapy (CCRT)-induced oral mucositis. It was via improving their immunity as they experienced a decreased reduction rate of CD3+, CD4+, and CD8+ T cells compared to the placebo group [53]. Importantly, they figured out that the probiotic cocktail altered the composition of the intestinal microbiome in patients with NPC, in which it increased the abundance of *Firmicutes* and decreased the abundance of *Bacteroidetes* and *Actinobacteria* to normal levels. As a result, the probiotic cocktail restored the gut dysbiosis in patients with NPC who underwent CCRT, resulting in the attenuation of CCRT-induced oral mucositis severity. For the rat mucositis model, both radiation and chemotherapy were used for the induction of oral mucositis with busulfan. There was a significant decrease in the mRNA expression of proinflammatory cytokines IL-6, IL-1β, and TNF in the tongue tissue, indicating that the probiotic cocktail reduced tongue tissue inflammation and pathological damage in the rats with oral mucositis caused by radiotherapy and chemotherapy. Also, the probiotic cocktail downregulated the expressions of TLR4 and P-NF-κB/NF-κB, and significantly inhibited cell apoptosis. In addition, the probiotic cocktail significantly restored the intestinal tight junction proteins ZO-1 and Claudin-1expression to normal levels. Thus, immune activation and inflammation are prevented, reducing TNF, IL-1β, and IL-6 in the oral cavity, which eventually could ameliorate oral mucositis [53]. Finally, the results revealed that the probiotic cocktail could reverse the gut dysbiosis induced by radiotherapy and chemotherapy in rats as well as by increased beneficial bacteria like *Firmicutes*, *Lachnospiraceae*, and *Ruminococcus*, while reducing harmful bacteria like *Bacteroidetes* and *Bacteroides*, suggesting that it helps prevent inflammation in the treated rats. Together, the probiotic cocktail significantly reduced the severity of oral mucositis in patients with NPC, possibly by regulating gut microbiota and boosting immunity. Experiments on rats further confirmed that the probiotic cocktail alleviates CCRT-induced oral mucositis severity by modulating gut dysbiosis related to inflammatory responses.

### 6.3. The Strengths, Gaps in the Knowledge, and Suggested Improvements

It was very insightful of the authors to discuss the modulation of the oral and intestinal microbiota in terms of ameliorating oral mucositis, summarized in Table 2, as it is a relatively easy and uncomplicated approach for the patients. However, in the study conducted by Xiao et al. (2021) [52], the authors should have provided some information on how the oral microbiota transplantation approach is performed for patients in practice. In this context, while microbiome-targeted interventions, including probiotics and oral microbiota transplantation, demonstrate promising therapeutic potential in reducing the severity of CRIOM, the current evidence remains limited. Clinical data are derived from relatively small and specific patient cohorts, whereas mechanistic insights are largely supported by preclinical models. Consequently, issues related to study design, sample size, and translational applicability should be carefully considered when interpreting these findings, and further well-designed clinical studies are required to validate their clinical utility.

Despite promising preclinical findings, the feasibility and safety of oral microbiota transplantation remain insufficiently characterized, as key aspects such as donor screening, microbial preparation, and delivery methods are not yet standardized and require further investigation in clinical settings [52]. In addition, the identification of suitable super donors (i.e., individuals with a stable, health-associated microbiome capable of conferring therapeutic benefit), the need for rigorous screening protocols, and logistical and ethical considerations related to donor selection and transplantation procedures further complicate clinical implementation [54].

Finally, microbiota manipulation may lead to alterations in microbial composition, as demonstrated by clinical and translational studies showing shifts in specific bacterial taxa following intervention, which may in turn disrupt host–microbiome homeostasis, trigger immune responses, and, given the complex and dynamic nature of the microbiota influenced by multiple host and environmental factors, result in outcomes that are difficult to predict [55,56,57].

## 7. Conclusions

To date, numerous strategies have been explored to prevent or mitigate oral mucositis, and the presented therapeutic approaches highlight selected studies that exemplify different biological mechanisms, including anti-inflammatory and microbiome-modulating interventions. Cytokine toxicity and microbiota dysbiosis represent two key contributors to CRIOM in head and neck cancer patients. The implemented strategies demonstrated significant efficacy in managing severe oral mucositis conditions, showcasing their potential as viable treatments for mitigating the intensity and discomfort associated with this condition. This comprehensive review of the currently known approaches identified a range of viable strategies to alleviate the painful and debilitating effects of oral mucositis for patients. Given the multifactorial nature of CRIOM, integrative strategies targeting both inflammatory pathways and microbiota dysbiosis may offer enhanced therapeutic benefit and represent a promising direction for future research.

However, further examination of numerous parameters, including sex and age, is still required to ascertain the feasibility of these approaches. Sex and age represent important biological variables that may influence the pathogenesis and severity of CRIOM in patients undergoing chemotherapy and radiotherapy for head and neck cancer. Sex-related differences in immune responses, molecular profiles, and pharmacokinetics have been shown to affect both treatment efficacy and toxicity, potentially contributing to variability in mucosal injury and inflammatory responses following therapy [58]. In head and neck cancer, real-world data suggest that female patients may exhibit improved survival outcomes compared to males across different therapeutic regimens, although these findings remain variable and influenced by confounding factors [59]. In addition, aging is associated with alterations in treatment tolerance, symptom burden, and functional status during radiotherapy, which may impact mucosal damage, healing capacity, and overall inflammatory response [60]. Clinical evidence further indicates that, in selected elderly patients receiving chemoradiotherapy, treatment adherence and toxicity profiles can be comparable to those of younger individuals, although comorbidities and increased susceptibility to treatment-related complications remain important considerations [61]. Consistent with these observations, salivary cytokine levels, including IL-1β, IL-6, IL-8, and TNF-α, have been shown to increase with age and are associated with oral inflammation, while sex-related differences appear limited, with only modest variations reported [62]. Together, these findings suggest that sex- and age-related biological differences may influence both the development and resolution of CRIOM; however, their precise mechanistic roles remain incompletely understood and warrant further investigation. The meticulous evaluation will ensure that the proposed methods are effective and applicable across diverse patient populations.

It is important to acknowledge that commonly used preclinical models, particularly rodent models, may not fully replicate the complexity of human oral mucosa. Differences in epithelial structure, immune responses, and microbiome composition between animals and humans may influence disease progression and treatment responses. Importantly, even in vitro models using human gingival fibroblasts, although more physiologically relevant than animal-derived systems, remain limited in their ability to replicate the complex in vivo microenvironment of the oral mucosa. These models do not capture the dynamic interactions among epithelial, immune, and stromal cells, nor do they account for microbiome-related influences that are critical in CRIOM pathogenesis. Therefore, while these models provide valuable mechanistic insights, caution is required when translating these findings to clinical settings.

In light of these limitations, while the studies summarized in this review highlight promising therapeutic strategies for the management of CRIOM, the majority of the available evidence is derived from preclinical models, including in vitro and animal studies. Although limited translational studies and a randomized clinical trial provide encouraging results, the current body of evidence remains insufficient to support widespread clinical implementation. Therefore, further well-designed, large-scale clinical trials are required to validate these approaches and facilitate their translation into clinical practice. In this context, these findings should be interpreted in the context of current clinical practice, where CRIOM management includes preventive oral care and oral hygiene measures, pain control, and selected interventions such as low-level laser therapy, while the approaches discussed here remain largely investigational.

## Figures and Tables

**Table 1:** Summary of anti-inflammatory approaches alleviating chemotherapy- and/or radiotherapy-induced oral mucositis.

Treatment	Mechanism	Type of Experiment	Model	Sample Size	Outcome	Key Limitation/Level of Evidence	Intervention Type	Reference
Pentoxifylline, Atorvastatin, Trans-Caryophyllene	↓ TNF, ↓ IFN-γ, ↓ TGF-β; reduced inflammation; enhanced re-epithelialization	Preclinical in vivo study (animal model)	Wistar rats, 5-FU-induced OM	*n* = 32 rats (4 groups, *n* = 8 per group)	Attenuation of Oral Mucositis	Preclinical (animal model); limited clinical translation	Preventive	[33]
Azilsartan (AZT)	Angiotensin II blockade; ↓ TNF, ↓ IL-1β; ↑ IL-10, ↑ growth factors (VEGF, FGF, KGF, TGF-α)	Preclinical in vivo study (animal model)	Syrian hamsters, 5-FU-induced OM	*n* = 36 hamsters (6 groups, *n* = 6 per group)	Attenuation of Oral Mucositis	Preclinical (animal model); limited clinical translation	Preventive	[34]
Recombinant Human IL-11 (rhIL-11)	↓ IL-1β, ↓ TNF expression; reduced mucosal cytokine load	Preclinical in vivo study (animal model)	Golden Syrian hamsters, radiation-induced OM	*n* = 30 hamsters (2 groups, *n* = 15 per group)	Attenuation of Oral Mucositis	Preclinical (animal study); limited clinical translation	Preventive	[35]
Low-Level Laser Therapy (LLLT)	↓ TNF, ↓ IL-6, ↓ IL-8 expression/synthesis	In vitro study (primary human cell model)	Primary cells from *n* = 3 donors (replicated experiments)	Primary cells from *n* = 3 donors (replicated experiments)	Attenuation of Oral Mucositis	In vitro study; limited clinical translatability	Therapeutic	[36]

**Table 2:** Summary of microbiome-modulating approaches alleviating chemotherapy- and/or radiotherapy-induced oral mucositis.

Treatment	Mechanism	Type of Experiment	Model	Sample Size	Outcome	Key Limitation/Level of Evidence	Intervention Type	Reference
Oral Microbiota Transplantation (OMT)	Restores microbial diversity; ↓ IL-1, IL-6, TNF, TGF-β; ↑ S100a9 promoting repair	Translational study (preclinical + clinical data)	Mouse model + patients with head and neck cancer	Animal study: *n* = 90 mice (4 groups); Clinical cohort: *n* = 44 patients	Attenuation of Oral Mucositis	Primarily preclinical; limited clinical validation	Therapeutic	[52]
Modified Probiotic Cocktail (*L. plantarum*, *B. animalis*, *L. rhamnosus*, *L. acidophilus*)	Restores gut–oral microbiome; ↓ NF-κB, TLR4, IL-1β, IL-6, TNF; ↑ barrier proteins (ZO-1, Claudin-1), ↑ T-cells	Phase II randomized, double-blind, placebo-controlled clinical trial + preclinical in vivo study	NPC patients (CCRT) and the busulfan rat model	Clinical trial: *n* = 70 patients; Animal study: *n* = 39 rats	Attenuation of Oral Mucositis	Strong translational evidence; limited to single-center Phase II trial	Preventive	[53]

## Data Availability

No new data were created or analyzed in this study.
